# T1 mapping across the ejection fraction spectrum

**DOI:** 10.1186/1532-429X-18-S1-P8

**Published:** 2016-01-27

**Authors:** Bashir Alaour, Paul Haydock, David Gelder, Stephen Harden, James Shambrook, Charles Peebles, Jennifer Bryant, Andrew S Flett

**Affiliations:** grid.430506.4University Hospital Southampton NHS Foundation Trust, Southampton, UK

## Background

T1 mapping is a rapidly evolving field and is emerging as a novel quantitative tissue characterisation technique with potential applications in fibrosis, oedema, fat, iron and other patho-physiology. Its significance in heart failure is not yet fully explored but has the potential to add insights into disease processes and aid in refining diagnosis.

## Methods

242 consecutive consenting patients referred for CMR between December 2014 and June 2015 were scanned on a 1.5T Magnetom Avanto (Siemens Healthcare, Erlangen) using a MOLLI (WIP#448, 5:3:3 acquisition scheme, motion correction and automatically generated T1 map). In all cases on the basal short axis slice, a single, slender ROI was placed in the mid wall of the septum with meticulous attention to avoid partial volume of blood/and or scar areas to derive the native T1 in msec. The patients were grouped by ejection fraction: <45%, 45-55% and >55%. 15 healthy volunteers were also used as a control group. Ejection fraction (EF), LV volumes, LV mass and BSA indexed left atrial area (LAAi) were also acquired. Comparison was performed using ANOVA and linear regression with adjustment for demographic variables that were found to vary between groups (age and BSAi LV mass).

## Results

**Figure 1:**
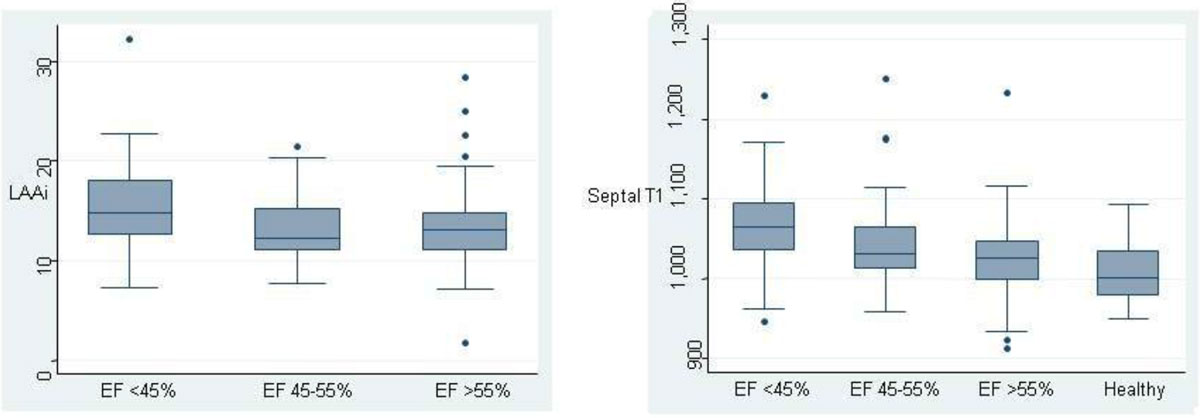
Figure 1

Baseline characteristics are given in the table. Unadjusted LAAi values were similar in the EF 44-55% and EF >55% groups (13.3 ± 3.5) but significantly higher in the EF <45% group (15.5 ± 4.6, p = 0.009), see figure 1. After adjustment for confounding variables, LAAi did not vary between groups (14.2, 13.8 and 13.6 respectively) p = 0.81. For septal T1, the adjusted and unadjusted analyses showed a significant difference between the 4 groups, with an inverse relationship between T1 value and EF as grouped in the study.

On pair wise comparisons between groups, there was no difference in T1 values between the <45% and 45-55% group in all analyses. The T1 difference between the 45-55% and >55% groups was significant only after adjusting for age, sex and LV mass p = 0.04. The control group septal T1 significantly varied from both the <45% and 45-55% groups (p => 0.001 and p = 0.03 respectively after adjusting for age and sex). There was no difference between the Septal T1 between the control group and the >55% group (p = 0.9 after adjusting for age and gender).

## Conclusions

In a large cohort of consecutive patients, we have demonstrated that native T1 is prolonged in patients with low EF. Based on prior study, this is likely to reflect diffuse fibrosis. In addition we have shown that Native T1 is not different between patients referred for CMR with normal EF and healthy volunteers.

**Table 1:** Table 1

Variable	EF <45% (n = 35) n (%)	EF 45-55% (n = 44) n (%)	EF >55% (n = 163) n (%)	Controls (n = 15) n (%)	P-value
Gender: Female Male	14 (32%) 30 (68%)	10 (30%) 23 (70%)	74 (45%) 89 (55%)	9 (60%) 6 (40%)	0.09
	Mean (SD)	Mean (SD)	Mean (SD)	Mean (SD)	
Age	68.0 (10.3)	56.6 (17.6)	57.5 (17.7)	48.0 (13.4)	<0.001
BMI	27.1 (4.9)	27.9 (4.4)	28.1 (4.8)	-	0.50
BSA	1.95 (0.24)	1.99 (0.22)	1.95 (0.22)	-	0.70
LVmassi (g)	85.5 (23.5)	61.4 (13.3)	62.7 (17.8)	-	<0.001
Septal T1	1065(51)	1045(59)	1023(42)	1006+/-36	<0.001
Adjusted Septal T1	1066*	1047*	1023*	1005**	<0.001
	Median (IQR)	Median (IQR)	Median (IQR)		
End Diastolic Volume	197 (162, 248)	154 (130, 182)	132 (112, 159)	-	<0.001

*adjusted for age, gender and LVmassi. **adjusted for age and gender

